# The cellular and molecular etiology of the craniofacial defects in the avian ciliopathic mutant *talpid^2^*

**DOI:** 10.1242/dev.105924

**Published:** 2014-08

**Authors:** Ching-Fang Chang, Elizabeth N. Schock, Elizabeth A. O'Hare, Jerry Dodgson, Hans H. Cheng, William M. Muir, Richard E. Edelmann, Mary E. Delany, Samantha A. Brugmann

**Affiliations:** 1 Division of Plastic Surgery, Department of Surgery, Cincinnati Children's Hospital Medical Center, Cincinnati, OH 45229, USA; 2 Division of Developmental Biology, Department of Pediatrics, Cincinnati Children's Hospital Medical Center, Cincinnati, OH 45229, USA; 3 College of Agricultural and Environmental Sciences, Department of Animal Science, University of California Davis, Davis, CA 95616, USA; 4 Microbiology and Molecular Genetics, Michigan State University, East Lansing, MI 48824, USA; 5 USDA Avian Disease and Oncology Laboratory, East Lansing, MI 48823, USA; 6 Department of Animal Sciences, Purdue University, West Lafayette, IN 47907, USA; 7 Center for Advanced Microscopy and Imaging, Miami University, Oxford, OH 45056, USA

**Keywords:** Primary cilia, Craniofacial, *talpid^2^*, Gli processing, Hedgehog signaling, Ciliopathies, Chicken

## Abstract

*talpid^2^* is an avian autosomal recessive mutant with a myriad of congenital malformations, including polydactyly and facial clefting. Although phenotypically similar to *talpid^3^*, *talpid^2^* has a distinct facial phenotype and an unknown cellular, molecular and genetic basis. We set out to determine the etiology of the craniofacial phenotype of this mutant. We confirmed that primary cilia were disrupted in *talpid^2^* mutants. Molecularly, we found disruptions in Hedgehog signaling. Post-translational processing of GLI2 and GLI3 was aberrant in the developing facial prominences. Although both GLI2 and GLI3 processing were disrupted in *talpid^2^* mutants, only GLI3 activator levels were significantly altered in the nucleus. Through additional fine mapping and whole-genome sequencing, we determined that the *talpid^2^* phenotype was linked to a 1.4 Mb region on GGA1q that contained the gene encoding the ciliary protein C2CD3. We cloned the avian ortholog of *C2CD3* and found its expression was ubiquitous, but most robust in the developing limbs and facial prominences. Furthermore, we found that C2CD3 is localized proximal to the ciliary axoneme and is important for docking the mother centriole to the ciliary vesicle and cell membrane. Finally, we identified a 19 bp deletion in *talpid^2^ C2CD3* that produces a premature stop codon, and thus a truncated protein, as the likely causal allele for the phenotype. Together, these data provide insight into the cellular, molecular and genetic etiology of the *talpid^2^* phenotype. Our data suggest that, although the *talpid^2^* and *talpid^3^* mutations affect a common ciliogenesis pathway, they are caused by mutations in different ciliary proteins that result in differences in craniofacial phenotype.

## INTRODUCTION

The chick is a classic embryological system that has been meticulously documented and described (Hamburger and Hamilton, 1951). Several institutions have bred and maintained mutant avian lines that have lent a significant amount of insight into numerous developmental processes (Robb et al., 2011). Some of the most well-studied avian genetic developmental mutants have been the talpids (*talpid*, *talpid^2^* and *talpid^3^*): three independently discovered, naturally occurring, autosomal recessive, lethal mutants characterized by pre-axial polydactyly, craniofacial anomalies and a host of other developmental defects (Abbott et al., 1959; Ede and Kelly, 1964a,b; MacCabe and Abbott, 1974). Although *talpid* became extinct, the study of the genetic and molecular etiology of the *talpid^2^* and *talpid^3^* phenotypes continues.

*talpid^2^* and *talpid^3^* have similar limb phenotypes, yet the craniofacial phenotypes are distinct. *talpid^3^* has severe hypotelorism, a hypoplastic frontonasal mass superior to the eyes, medially fused nasal placodes, a narrow and peg-like lower beak, and an oral cavity that is divided in two by the maxillary prominences fusing across the midline (Abbott et al., 1959, 1960; Ede and Kelly, 1964a; Hinchliffe and Ede, 1968; Buxton et al., 2004). By contrast, the *talpid^2^* facial phenotype is characterized by a short yet wide frontonasal prominence, bilateral clefting between the frontonasal and lateral nasal prominence and hypoglossia/aglossia (Schneider et al., 1999; Brugmann et al., 2010). Molecularly, the *talpid^3^* mutant has been more extensively studied and attributed to disruptions in Sonic Hedgehog (SHH) signaling (Buxton et al., 2004; Davey et al., 2006, 2007). Whereas the defect in SHH signaling was clear, understanding the molecular mechanism for the phenotype was confounded by the fact that the facial phenotype was indicative of a loss of SHH function, but the limb phenotype was indicative of a gain of SHH function (Buxton et al., 2004; Davey et al., 2006, 2007). A deeper understanding of the molecular etiology of the mutant was gained when the causal genetic element for *talpid^3^* was identified and characterized as a protein (now called TALPID3 after the mutant itself) that localizes to the basal body of the primary cilia (Yin et al., 2009).

Primary cilia are ubiquitous organelles that serve as a cellular hub for the transduction of numerous signaling pathways. Most notably, cilia have been identified as the location for post-translational processing of GLI proteins (Goetz and Anderson, 2010). GLI proteins are transcription factors that regulate a number of cellular processes (e.g. proliferation, specification and differentiation). Full-length GLI (GLIFL) can be processed via phosphorylation into the activator isoform (GLIA) or cleaved into the repressor isoform (GLIR) (Liu et al., 2005). Inhibition of GLI processing prevents production of GLIA and GLIR isoforms (Haycraft et al., 2005). Thus, an essential role of primary cilia is to establish the ratio of GLIA to GLIR proteins, which in turn controls transcription of SHH target genes.

Our earlier studies regarding the molecular mechanism of *talpid^2^* suggested defects in primary cilia (Brugmann et al., 2010); however, these studies did not address the extent of ciliary dysfunction, molecular consequences of aberrant cilia or the genetic cause of the mutation. Studies from other groups have suggested that the polydactylous phenotype of *talpid^2^* was attributable to constitutive activation of SHH signaling, in which activation of the SHH pathway occurs in the absence of a SHH ligand (Caruccio et al., 1999). The limb phenotype in the *talpid^2^* mutant has been attributed to defects in GLI processing (Caruccio et al., 1999), but an in-depth analysis of GLI protein expression in the facial prominences has not been explored.

No direct comparison or complementation analysis of *talpid^2^* and *talpid^3^* mutants has been undertaken. The similar phenotypes of the mutants raised the possibilities that the causative genetic elements for these mutants were: (1) alleles of the same gene with different grades of expression (Ede and Kelly, 1964b); (2) separate genetic insults within the same pathway; or (3) completely separate defects. Herein, we explore the cellular and molecular basis for the *talpid^2^* craniofacial mutation, comparing our findings with those in the *talpid^2^* limb and in *talpid^3^*. Furthermore, we use whole-genome sequencing of inbred *talpid^2^* lines to identify the causative locus for the *talpid^2^* mutation and confirm that it is distinct from the *talpid^3^* mutation.

## RESULTS

### *talpid^2^* embryos have craniofacial defects

**Fig. 1.**
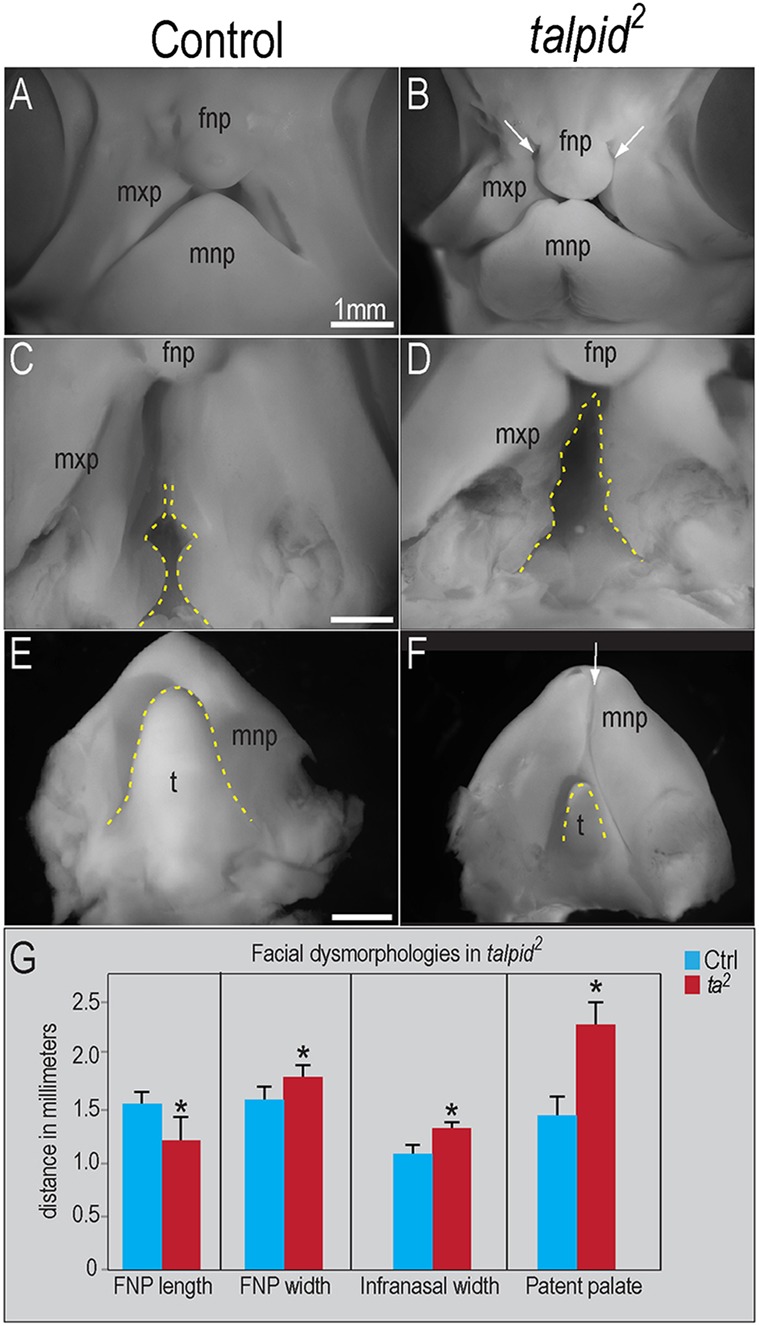
**Growth and development of the facial prominences is disrupted in *talpid^2^* mutants.** Day 7 control (*n*=10) and *talpid^2^* (*n*=4) embryos. (A,B) Frontal views of facial prominences; frontonasal (fnp), maxillary (mxp) and mandibular prominences (mnp). Bilateral clefting in *talpid^2^* embryos (white arrows in B). (C,D) Palatal views show increased patency of both the primary and secondary palate of *talpid^2^* embryos (dotted yellow line). (E,F) Dorsal view of the mnp. The mnp of *talpid^2^* embryos is clefted (white arrow in F) and exhibits hypoglossia (dotted yellow line). (G) Quantitative analysis of fnp and mxp growth. For fnp length, *P<0.02; for fnp width, *P<0.02; for infranasal width, *P<0.04; for patent palate, *P<0.02. t, tongue. Data are mean±s.e.m. Scale bars: 1 mm (A-F).

Although craniofacial defects have been previously reported in *talpid^2^* embryos (Abbott et al., 1959; Yee and Abbott, 1978; Caruccio et al., 1999; Schneider et al., 1999; Brugmann et al., 2010), an in-depth analysis of the development of facial prominences has not been reported. We examined control and *talpid^2^* embryos and analyzed the growth of the frontonasal, maxillary and mandibular prominences (Fig. 1). *talpid^2^* embryos displayed bilateral clefting between the frontonasal, lateral nasal and maxillary prominences (Fig. 1A,B). The frontonasal prominence was significantly shorter and wider in *talpid^2^* embryos relative to controls (Fig. 1A,B,G). Growth of the maxillary prominence was also disrupted. The maxillary prominences fuse to form the secondary palate. In avians, part of the secondary palate remains patent (Lillie, 1908) (Fig. 1C). In contrast to control embryos, the extent of patency was significantly increased in *talpid^2^* embryos (Fig. 1C,D,G). We also measured inner intercanthal distance and the width of the lateral nasal prominence. Neither of those measurements showed significant variation between control and *talpid^2^* embryos (data not shown). Thus, we conclude that the predominant cause of clefting in the *talpid^2^* embryos is the aberrant growth of the frontonasal and maxillary prominences. Finally, we analyzed the growth of the mandibular prominence. The mandibular prominences of *talpid^2^* embryos fail to fuse completely (Fig. 1E,F). In addition, a large percentage of *talpid^2^* embryos (10/12; 83%) exhibit hypoglossia/aglossia (Fig. 1E,F). These data confirm that the growth and development of the facial prominences are significantly disrupted in *talpid^2^* embryos.

### Primary cilia are disrupted throughout the *talpid^2^* embryo

**Fig. 2.**
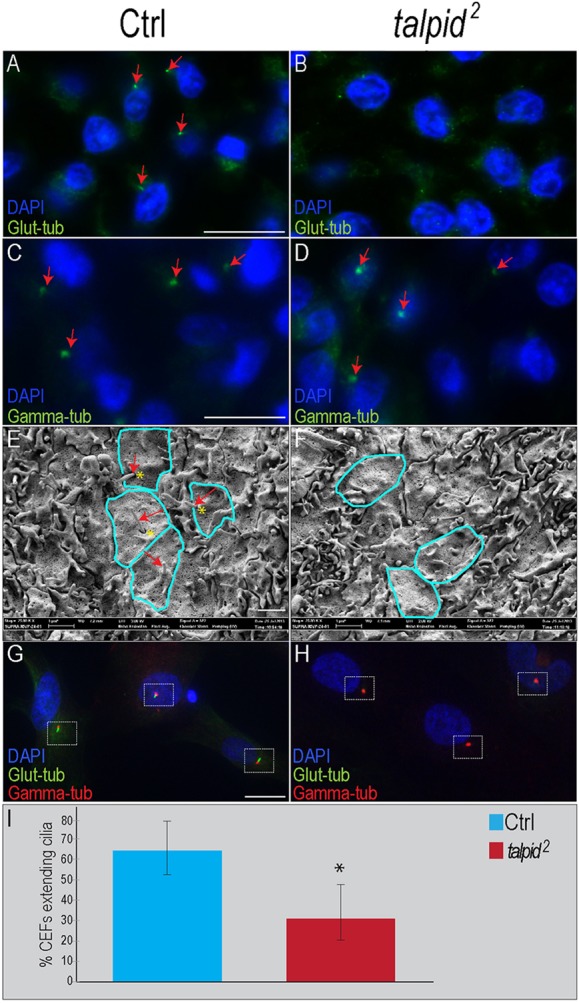
**Primary cilia are disrupted in *talpid^2^* mutants.** (A-D) Immunostaining for ciliary markers on facial sections. (A,B) Anti-glutamylated-tubulin in facial mesenchyme. Red arrows mark axoneme. (C,D) Anti-γ-tubulin in facial mesenchyme. Red arrows mark centriole. (E,F) Scanning electron microscopy of the ventricular surface of the neuroectoderm. Blue lines outline cells; red arrow indicates axoneme; yellow asterisks mark ciliary pockets. (G,H) Double glutamylated- and γ-tubulin immunostaining in CEFs. (I) Quantification of control (*n*=686) and *talpid^2^* (*n*=522) CEFs extending primary cilia; *P<0.005. Data are mean±s.e.m. Scale bars: 10 µm in A-D; 1 µm in E,F; 20 µm in G,H.

The *talpid^3^* phenotype had previously been shown to be a product of disrupted primary cilia (Yin et al., 2009). Our previous work identified disrupted cilia in *talpid^2^* mutants (Brugmann et al., 2010). We confirmed this finding by performing immunostaining with glutamylated-tubulin on facial mesenchyme (Fig. 2A,B). Glutamylated-tubulin staining in control avian embryos is localized to the ciliary axoneme (Fig. 2A), whereas staining in the *talpid^2^* mesenchyme is diffuse and unorganized (Fig. 2B). We also examined γ-tubulin staining in the centrosome (Fig. 2C,D). We found abundant, punctate γ-tubulin staining in the mesenchyme of control embryos (Fig. 2C). Although we were able to detect γ-tubulin in *talpid^2^* embryos (Fig. 2D), some staining was diffuse and not as punctate as in control embryos (Fig. 2D). To further characterize ciliary extension in the *talpid^2^* mutants, we performed scanning electron microscopy in the forebrain (Fig. 2E,F). We found numerous ciliary extensions within a ciliary pocket (Fig. 2E) in control embryos. In *talpid^2^* embryos, however, the majority of cells did not extend a cilium (Fig. 2F). Finally, we isolated and cultured chicken embryonic fibroblasts (CEFs) from both control and *talpid^2^* embryos and quantified the percentage of cells extending a cilium. Sixty-four percent of control CEFs extended a glutamylated-tubulin-positive extension adjacent to a punctate domain of γ-tubulin staining (Fig. 2G,I). By contrast, only 32% *talpid^2^* CEFs extended a glutamylated-tubulin-positive extension adjacent to a punctate domain of γ-tubulin staining (Fig. 2H,I). These results, in conjunction with our previous findings, strongly suggest that the *talpid^2^* phenotype, like the *talpid^3^* phenotype, is caused by a ciliary defect.

### SHH expression and activity are affected in the developing face and brain of *talpid^2^* embryos

**Fig. 3.**
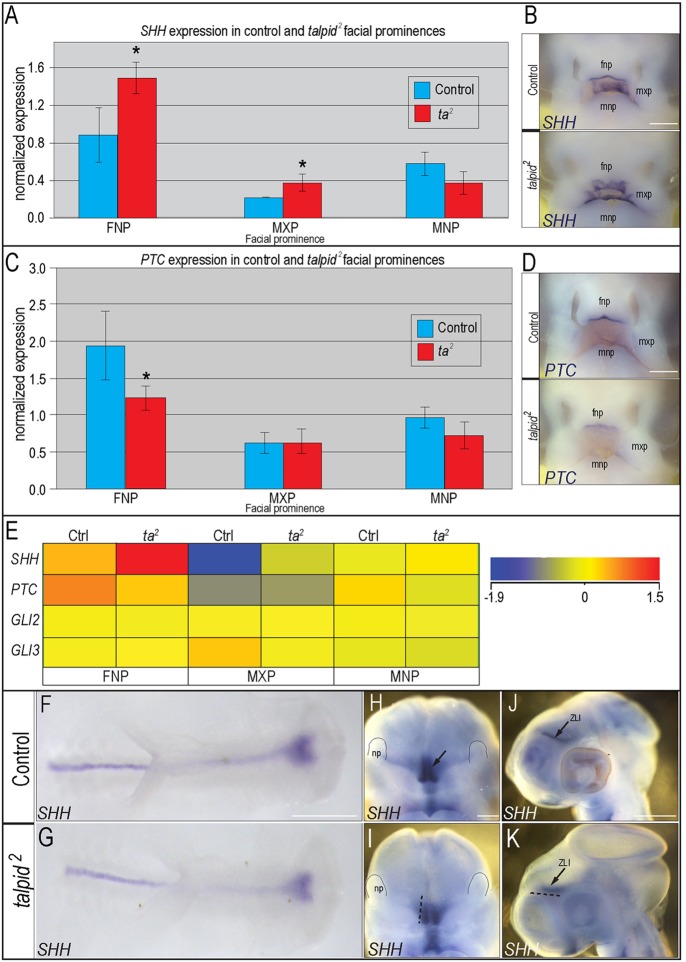
***SHH* signaling is disrupted in the developing face and brain of *talpid^2^* mutants.** (A) qRT-PCR for *SHH* in frontonasal (FNP), maxillary (MXP) and mandibular (MNP) prominences in control and *talpid^2^* embryos; *P<0.05. Data are mean±s.e.m. (B) *In situ* hybridization for *SHH* in control (*n*=7) and *talpid^2^* (*n*=3) HH25 embryos. (C) qRT-PCR for *PTC*; *P<0.03. Data are mean±s.e.m. (D) *In situ* hybridization for *PTC* in control (*n*=7) and *talpid^2^* (*n*=3) HH25 embryos. (E) RNA-seq results for *SHH*, *PTC*, *GLI2* and *GLI3* expression. (F-K) *In situ* hybridization for *SHH* in control and *talpid^2^* embryos. (F,G) Dorsal view of control (*n*=11) and *talpid^2^* (*n*=9) HH10 embryos. (H-K) Frontal and lateral views of control (*n*=8) and *talpid^2^* (*n*=4) HH20 embryos. Dotted black lines in I,K represent the size of the expression domain in the control embryo (H,J). ZLI, zona limitans intrathalamica. Scale bars: 250 µm in B,D; 600 µm in F,G; 100 µm in H,I; 200 µm in J,K.

Previous studies have reported aberrant *SHH* expression in *talpid^2^* embryos (Caruccio et al., 1999; Schneider et al., 1999; Agarwala et al., 2005)*.* As ciliary defects have been known to affect various tissues and developmental domains differently, and because previous reports suggest a gain of SHH function in the limb with a loss of SHH function in the face of *talpid^3^* (Buxton et al., 2004; Davey et al., 2006, 2007), we examined *SHH* expression and pathway activity in both the developing facial prominences and the developing brain. Quantitative RT-PCR (qRT-PCR) of individual facial prominences demonstrated that levels of *SHH* ligand expression varied in a prominence-specific manner. *SHH* expression was increased in both frontonasal and maxillary prominences; however, there was a slight decrease in expression in the mandibular prominence (Fig. 3A). These results were confirmed with *in situ* hybridization for *SHH* in HH25 control and *talpid^2^* embryos (Fig. 3B). An increase in *SHH* ligand typically results in increased activity of the pathway, as marked by increased *PATCHED* (*PTC*) transcription. Despite increased *SHH* ligand expression, *PTC* expression was significantly reduced in the frontonasal prominence (Fig. 3C). Conversely, *PTC* expression was not significantly altered in either the maxillary or mandibular prominences (Fig. 3C). Variation in *PTC* expression was confirmed with *in situ* hybridization (Fig. 3D). Finally, RNA-seq was performed on individual facial prominences of both control and *talpid^2^* embryos (Fig. 3E). These data support both qRT-PCR and *in situ* data suggesting that, although *SHH* ligand expression is increased in the frontonasal and maxillary prominences, pathway activity (as determined by *PTC* expression) is not. These data suggest *SHH* expression is altered in a prominence-specific manner and that ligand expression does not necessarily correlate with canonical SHH pathway activity in *talpid^2^* embryos.

The developing brain strongly influences the developing face (Marcucio et al., 2005; Young et al., 2010; Chong et al., 2012). There are several SHH signaling centers in the brain that can influence growth of the craniofacial complex, including the SHH domain in the ventral neural tube, forebrain and the zona limitans intrathalamica (ZLI). To determine whether centers of SHH activity were disrupted in the brain of *talpid^2^* embryos, we examined the expression of *SHH* at various stages of development. At HH10, the *SHH* domain in the ventral floor plate was evident in both control and *talpid^2^* embryos (Fig. 3F,G). At HH20, however, *SHH* expression in both the forebrain and ZLI was significantly reduced (Fig. 3H-K). Thus, although initially established correctly, later *SHH* signaling domains are reduced in the developing brain of *talpid^2^* embryos.

### Post-translational processing of GLI2 is disrupted in the *talpid^2^* mutant

**Fig. 4.**
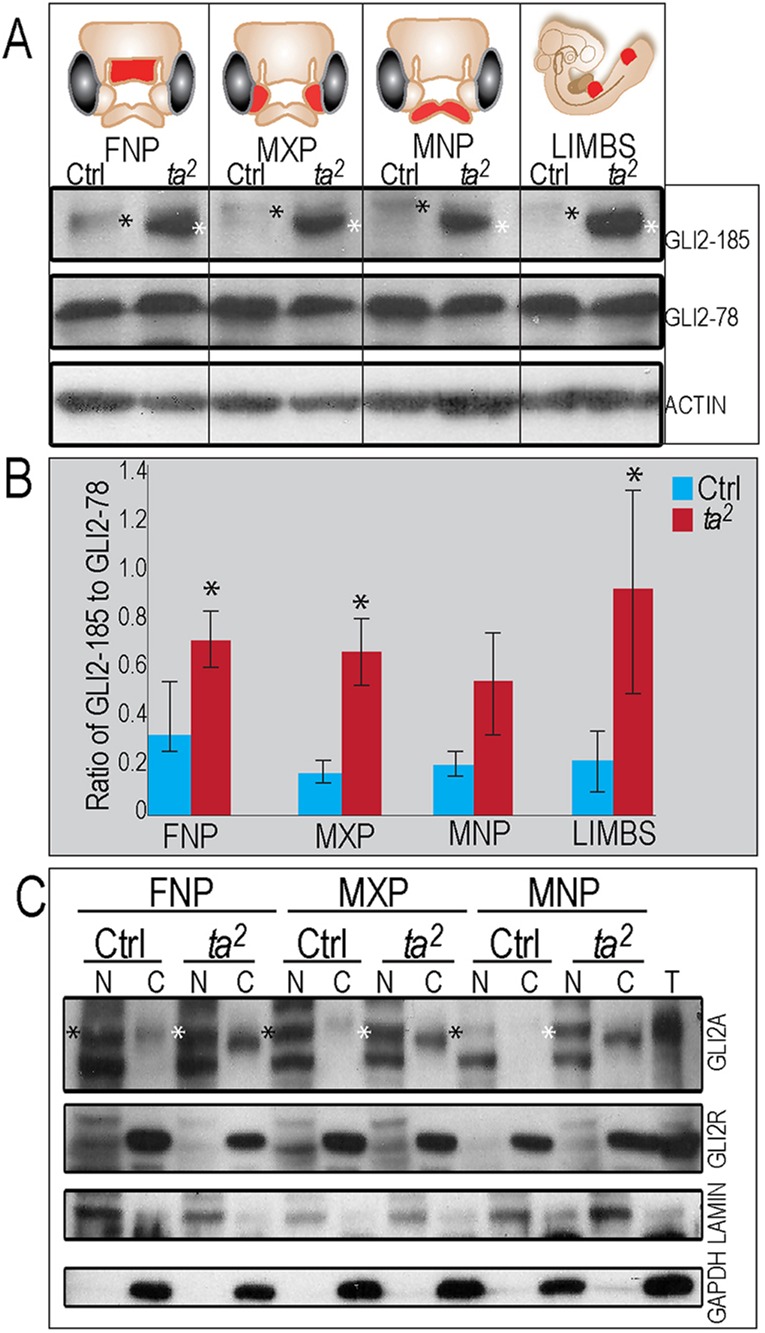
**Post-translational processing of GLI2 is disrupted in *talpid^2^* mutants.** (A) Western blot analysis for GLI2 in HH 25 facial prominences and limbs. Both full-length GLI2 protein (GLI2-185) and cleaved GLI2 (GLI2-78) are present. Gel mobility of GLI2-185 is higher in *talpid^2^* (compare black and white asterisks). (B) Ratio of GLI2-185 to GLI2-78 is increased in *talpid^2^.* *P<0.05. Data are mean±s.e.m. (C) Nuclear fractionation detects both GLI2 activator (GLI2A) and GLI2 repressor (GLI2R). LAMIN and GAPDH expression mark the nuclear (N) and cytosolic (C) compartment. T, total protein.

Functional primary cilia are required for the proper processing of full-length GLI proteins (GLIFL) into either a full-length activator or a cleaved repressor (Haycraft et al., 2005; Liu et al., 2005; May et al., 2005; Humke et al., 2010; Wen et al., 2010). To determine whether the *talpid^2^* craniofacial phenotype could be attributed to aberrant post-translational processing of the GLI transcription factors, we performed western blot analyses on individual facial prominences. We began our analysis with GLI2 because it functions as the primary activator of the SHH pathway (Ding et al., 1998; Motoyama et al., 1998; Park et al., 2000; Bai et al., 2002), and the limb phenotype in *talpid^2^* embryos is highly suggestive of a gain of SHH phenotype. Using an antibody that recognizes both full-length (GLI2-185) and cleaved isoforms (GLI2-78) of GLI2, we found significant disruptions in both the developing facial prominences and limbs (Fig. 4). Relative to control, there was a significant and consistent increase in the levels of GLI2-185 in all *talpid^2^* facial prominences and limb buds (Fig. 4A). Although GLI2 predominantly functions as a full-length isoform (Pan et al., 2006), we were able to detect an equivalent amount of the cleaved isoform in both control and *talpid^2^* tissue (Fig. 4A). The ratio of full-length versus cleaved activity is used as a measure of net SHH pathway activity (Huangfu and Anderson, 2005). The increase in level of GLI2-185 in *talpid^2^* mutants resulted in an increased ratio of GLI2-185 to GLI2-78, as quantified by densitometry (Fig. 4B).

To test whether the increased levels of GLI2-185 detected via whole-lysate western blot correlate with increased levels in the nuclear GLI2A in *talpid^2^* mutants, we performed nuclear fractionation analysis (Fig. 4C). Although we detected increased GLI2-185 in all prominences of the *talpid^2^* mutant (Fig. 4A,B), our nuclear fractionation result indicated that the levels of nuclear GLI2A were equivalent within control and *talpid^2^* mutants (Fig. 4C; supplementary material Table S1). Furthermore, we did not note any significant change in the amount of cleaved nuclear GLI2R in *talpid^2^* mutants (Fig. 4C; supplementary material Table S1). Taken together, our experiments indicate that, although full-length GLI2 processing is impaired in *talpid^2^* mutants, nuclear levels of GLI2A and GLI2R are not altered.

To function properly, GLI proteins require post-translational modifications, including phosphorylation (Pan et al., 2009; Canettieri et al., 2010; Cox et al., 2010; Hui and Angers, 2011). In light of our results showing an accumulation of *talpid^2^* full-length GLI2 with higher mobility than control full-length GLI2 (Fig. 4A) and multiple bands being detected in our nuclear fractionation (Fig. 4C), we tested the hypothesis that improper phosphorylation accounted for the changes in the mobility of GLI2-185 between control and *talpid^2^*. Phosphatase treatment did not alter mobility of control GLI2-185 to that of *talpid^2^* GLI2-185 (supplementary material Fig. S1A), suggesting that aberrant phosphorylation is not the cause of increased mobility of *talpid^2^* full-length GLI2. Regardless of increased mobility of *talpid^2^* GLI2-185, the mobility and overall amount of *talpid^2^* GLI2A protein in the nucleus was unchanged relative to control embryos and thus was determined not to be a possible cause for the *talpid^2^* phenotype.

### Post-translational processing of GLI3 is disrupted and GLI3 activator is increased in *talpid^2^* mutants

**Fig. 5.**
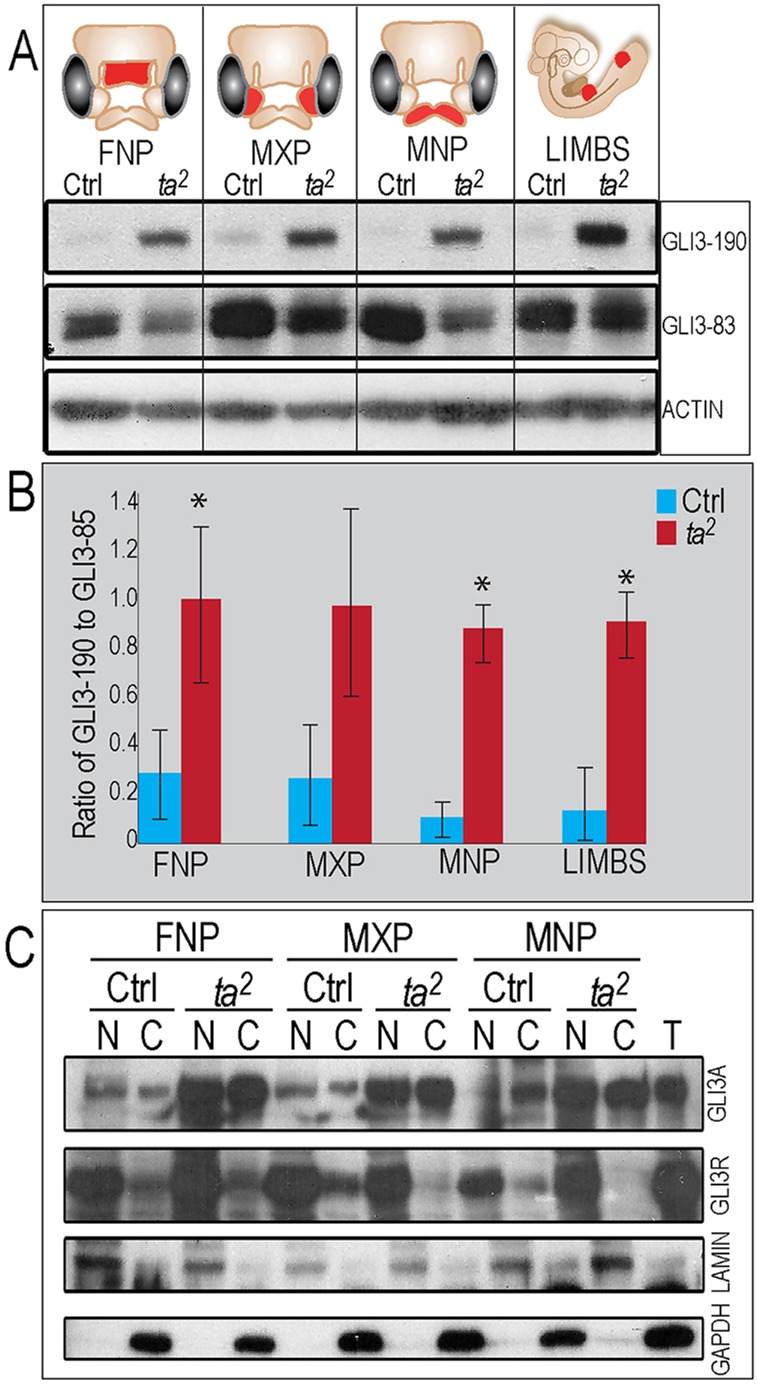
**Post-translational processing of GLI3FL is disrupted in *talpid^2^*.** (A) Western blot analysis for GLI3 in HH 25 facial prominences and limbs. Both full-length GLI3 protein (GLI3-190) and cleaved GLI3 (GLI3-83) are present. (B) Ratio of GLI3-190 to GLI3-83 is increased in *talpid^2^.* *P<0.05. (C) Nuclear fractionation detects both GLI3 activator (GLI3A) and GLI3 repressor (GLI3R). LAMIN and GAPDH expression mark the nuclear (N) and cytosolic (C) compartment. T, total protein.

We next examined the levels of GLI3 between control and *talpid^2^* facial prominences (Fig. 5A). Similar to what we observed with GLI2, we found an increase in the levels of full-length GLI3 (GLI3-190) in *talpid^2^* facial prominences and limb buds (Fig. 5A). Contrary to what we observed with the cleaved isoform of GLI2, levels of the cleaved GLI3 isoform (GLI3-83) were reduced in the *talpid^2^* facial prominences relative to controls (Fig. 5A). Quantification of gel images by densitometry indicated that the ratio of GLI3-190 to GLI3-83 was also increased in *talpid^2^* (Fig. 5B). Thus, our data suggest that in *talpid^2^*, GLI3 processing is also disrupted, resulting in an accumulation of GLI3-190 at the expense of GLI3-83.

Similar to GLI2, full-length GLI3 can be degraded in the cytoplasm or enter the nucleus as a full-length activator (GLI3A) or cleaved repressor (GLI3R). Contrary to GLI2, GLI3 acts as the predominant repressor of the SHH pathway (Vortkamp et al., 1992; Hui and Joyner, 1993; Biesecker, 1997; Persson et al., 2002). Nuclear fractionation revealed that increased levels of whole lysate GLI3-190 correlated with a robust increase of nuclear GLI3A in *talpid^2^* mutants (Fig. 5C; supplementary material Table S1). Furthermore, although GLI3-83 levels appeared slightly decreased via whole lysate examination, nuclear fractionation suggests that the total levels of GLI3R in the nucleus are equivalent between the control and *talpid^2^* embryos (Fig. 5C). Detection of increased nuclear GLI3A supports a hypothesis that increase of GLI3 activator activity is the cause of the *talpid^2^* facial phenotypes.

To determine the cause of aberrant GLI-190 production, we assessed the phosphorylation status of GLI3-190 in control and *talpid^2^* embryos using phosphatase treatment (supplementary material Fig. S1B). We did not observe differential gel mobility between control and *talpid^2^* GLI3-190. Although the amount of GLI3-190 was increased in *talpid^2^*, we observed similar shifts in mobility. These results indicate that aberrant phosphorylation is not the cause of increased full-length, nuclear GLI3A in *talpid^2^* mutants.

### Genetic mapping and identification of the *talpid*^2^ locus

**Fig. 6.**
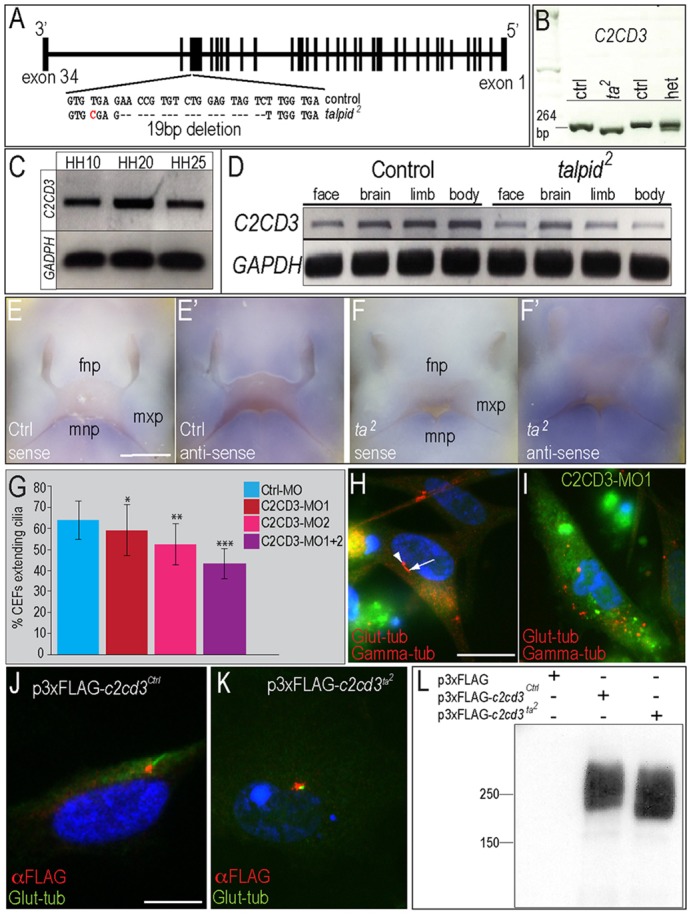
***C2CD3* is the candidate gene for the *talpid^2^* mutation.** (A) Chicken *C2CD3*. A 19 bp deletion was identified within exon 32 of *talpid^2^*. (B) RT-PCR analysis of *C2CD3* in control, *talpid^2^* and heterozygous embryos. (C) RT-PCR for *C2CD3* in whole embryos. (D) RT-PCR for *C2CD3* in various domains of HH25 embryos (*n*=3). (E-F′) *In situ* hybridization for *C2CD3.* (G) Quantification of CEFs extending cilia after transfection with a control-MO (*n*=342), C2CD3-MO1 (*n*=559), C2CD3-MO2 (*n*=551) or C2CD3-MO1+C2CD3-MO2 (*n*=573); *P<0.05, **P=0.001, ***P<0.0001. Data are mean±s.e.m. (H) Immunofluorescence marking glutamylated-tubulin (arrow) and γ-tubulin (arrowhead) on non-transfected CEFs. (I) Glutamylated-tubulin expression is lost on CEFs transfected with C2CD3-MO1. (J,K) Double immunofluorescence marking glutamylated-tubulin (green) and FLAG (red) on CEFs transfected with p3xFLAG-*C2CD3^Ctrl^* (J) or p3xFLAG-*C2CD3^ta2^* (K). (L) Western blot analysis of CEFs transfected with p3xFLAG-*C2CD3^Ctrl^* and p3xFLAG-*C2CD3^ta2^*. Scale bars: 500 µm in E-F′; 20 µm in H,I; 10 µm in J,K.

The *talpid^3^* phenotype was linked to a ciliary gene encoded on chromosome 5 (GGA5) (Yin et al., 2009). Using a 60K SNP array, we previously identified five candidate chromosome locations (two on GGA1q, two on GGA10q and one on GGA15), none of which mapped to the *talpid^3^* locus (Robb et al., 2011). Variation between the mutant talpid2.003 line and the congenic UCD003 control line was examined by whole-genome sequencing, and candidate genes and alleles were examined at all five intervals (data not shown; supplementary material Tables S2 and S3). These data indicated that the telomeric region of GGA1q (193.7 Mb to 195.2 Mb) exhibited at least a threefold higher ratio of *talpid^2^*-specific SNPs over any other interval (supplementary material Table S4), suggesting that this interval contained fixed, *talpid^2^*-specific polymorphisms derived from the original mutant line. Analysis of this interval suggested three potential candidate genes with variations that had potential to impair gene function: *C2CD3* (C2 calcium-dependent domain containing 3), *NUP98* (nucleoporin, 98 kDa) and *NEU3* (neuraminidase 3). We subsequently resequenced all three candidates from both control and mutant samples, and did not find any likely causal polymorphisms, significant changes in transcript size or changes in level of expression in *NUP98* and *NEU3* (data not shown). As demonstrated by our cDNA cloning and sequencing, the chicken ortholog of *C2CD3* produces a 7496 bp transcript with 34 exons (Fig. 6A; supplementary material Fig. S2). Whole-genome sequencing, cDNA sequencing and resequencing of the talpid2.003 line confirmed one insertion, one deletion and nine missense differences within the *C2CD3* open reading frame between control and *talpid^2^* genomes (supplementary material Table S5). The majority of the missense variants were likely to be tolerated and were located in regions of low complexity (according to protein modeling) with weak evolutionary conservation (data not shown); they were thus not likely to be causative. Moreover, subsequent resequencing of *talpid^2^* alleles in a commercial background line (rather than being congenic on UCD003, as talpid2.003 is) eliminated most of the missense mutations as potential causal alleles. The top candidate for the causal mutation was a 19 bp deletion in the 3′ end of exon 32 (Fig. 6A) that produces a premature stop codon leading to the deletion of amino acids 2210-2330. PCR analysis supported our sequencing results and detected the 19 bp deletion in *talpid^2^* mutants (Fig. 6B).

We next examined the temporal and spatial expression of chicken *C2CD3* in control and *talpid^2^* embryos. Temporally, we found *C2CD3* was robustly expressed throughout craniofacial development (Fig. 6C). To examine spatial expression, we isolated developing face, brain, limb and body from HH25 embryos (Fig. 6D). *C2CD3* was ubiquitously expressed throughout all dissected domains of control and *talpid^2^* embryos. Furthermore, we visualized *C2CD3* expression via *in situ* hybridization. Whereas we did not detect any expression with the sense probe (Fig. 6E,F), low levels of ubiquitous expression and higher levels of localized expression in the branchial arches and limbs were detected in both control and *talpid^2^* embryos (Fig. 6E′,F′; supplementary material Fig. S3). This expression pattern is consistent with *C2CD3* expression in murine embryos (Hoover et al., 2008) and consistent with the *talpid^2^* craniofacial and limb phenotype.

Next, to confirm that C2CD3 plays a role in ciliogenesis, we generated control and two distinct C2CD3-specific morpholinos. Control CEFs were transfected with a control morpholino, or with one or both C2CD3 morpholinos (Fig. 6G). Similar to non-transfected control CEFs (Fig. 2I), ∼63% of CEFs transfected with control MO extended a primary cilium (Fig. 6G). After transfection with C2CD3-MO1, C2CD3-MO2 or C2CD3-MO1 plus C2CD3-MO2, the number of CEFs extending cilia was significantly reduced (Fig. 6G). For example, the ciliary basal body and axoneme were clearly present in cells not transfected with C2CD3-MO; however, in the presence of C2CD3-MO, axoneme extension was absent (Fig. 6H,I). Taken together, these experiments suggest that *C2CD3* contains the causal allele for the *talpid^2^* mutant, that *C2CD3* is expressed in areas correlating with the *talpid^2^* phenotype and that MO-induced reduction of C2CD3 protein negatively affects ciliary extension.

C2CD3 is hypothesized to localize to either the proximal axoneme (transition zone) (Zhang and Aravind, 2012) or to the distal centriolar region (transition fibers) (Balestra et al., 2013; Ye et al., 2014). To determine where C2CD3 localized within avian cells, we transfected CEFs with FLAG-tagged control *C2CD3* (*C2CD3^Ctrl^*). We detected FLAG-*C2CD3^Ctrl^* adjacent to, but not overlapping with, the ciliary axoneme (Fig. 6J). These results support previous findings in humans and mice suggesting that C2CD3 localizes to the distal aspect of the centrioles within the transition fibers (Balestra et al., 2013; Ye et al., 2014). We next tested whether the 19 bp deletion in *talpid^2^* *C2CD3* affected the cellular localization of this protein. FLAG-*C2CD3^ta2^* was detected proximal to the axoneme, suggesting that the mutation does not affect the cellular localization of C2CD3 (Fig. 6K).

To determine whether the 19 bp deletion in *talpid^2^* *C2CD3* affected C2CD3 protein stability and size, we transfected both FLAG-*C2CD3^Ctrl^* and FLAG-*C2CD3^ta2^* into CEFs and performed western blot analysis. As expected, cells transfected with the FLAG-*C2CD3^ta2^* produced a significantly smaller protein relative to FLAG-*C2CD3^Ctrl^* (Fig. 6L), confirming that the 19 bp deletion generated a premature stop codon, producing a truncated version of C2CD3 in *talpid^2^* embryos. Furthermore, our western blot analysis confirms that the *talpid^2^* mutation does not affect C2CD3 stability or expression levels, as equal amounts of anti-FLAG staining were observed. Thus, although truncated, C2CD3 is still produced and properly localized in *talpid^2^* embryos.

### Ciliary vesicle docking and intracellular ciliogenesis is disrupted in *talpid^2^* embryos

**Fig. 7.**
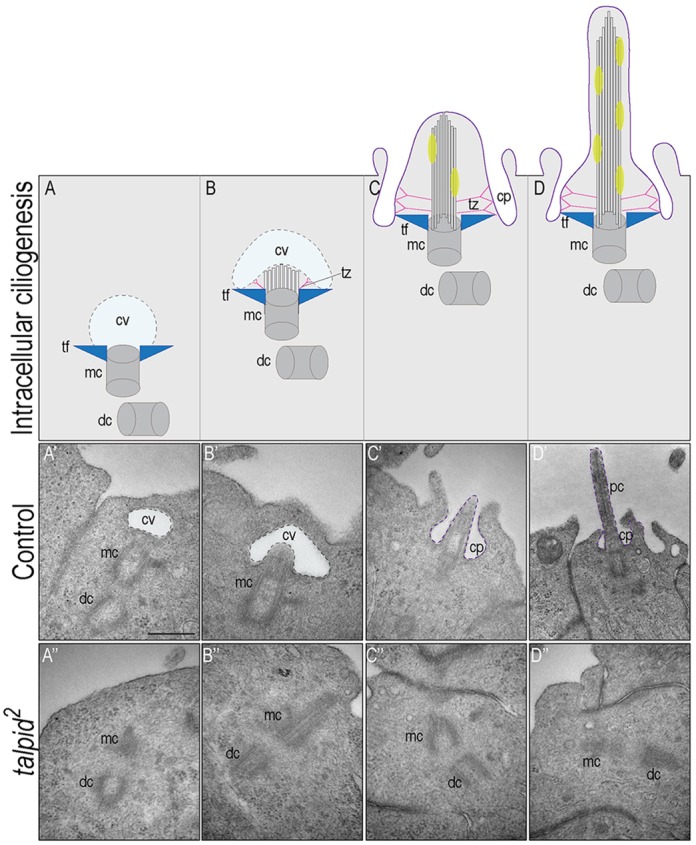
**Intracellular ciliogenesis is disrupted in *talpid^2^* embryos.** Hypothesized steps of intracellullar ciliogenesis. (A) A ciliary vesicle (cv) binds to the distal end of the mother centriole (mc), via associations with transition fibers (tf). (B) Microtubule and transition zone (tz) outgrowth emerges and invaginates the cv. (C) Docking of the centriolar/cv complex to the plasma membrane. (D) Axonemal outgrowth. (A′-D′) Steps of intracellular ciliogenesis in control embryos (*n*=11). (A″-D″) Intracellular ciliogenesis is significantly disrupted in *talpid^2^* embryos. Association of the mc with the cv and progression of the centriolar/cv complex docking with the plasma membrane rarely occur in *talpid^2^* embryos (*n*=15). dc, daughter centriole; pc, primary cilium; cp, ciliary pocket. Scale bars: 500 nm in A′-D″.

A recent study examining the role of C2CD3 in murine cells reported that the protein was localized within the transition fibers, it was required for initiating ciliogenesis via the recruitment of other centriolar distal appendage and intraflagellar transport proteins, and it was required for ciliary vesicle docking to the mother centriole (Ye et al., 2014). To determine whether the mechanism of avian C2CD3 function was similar to that of murine C2CD3, we analyzed control and *talpid^2^* embryos via transition electron microscopy (TEM). Specifically, we used TEM to examine intracellular ciliogenesis: the process of ciliogenesis that uses transition fibers to associate with a ciliary vesicle and creates a ciliary pocket to establish an extended primary cilium (Fig. 7A-D) (Molla-Herman et al., 2010; Reiter et al., 2012). Association of the mother centriole with a ciliary vesicle was clearly detected in ∼45% of control cells (Fig. 7A′). Furthermore, invagination of the ciliary vesicle associated with microtubule outgrowth from the mother centriole (Fig. 7B′), association of the mother centriole with the plasma membrane (Fig. 7C′) and formation of an extended cilium within a ciliary pocket (Fig. 7D′) were also frequently observed in control cells. By contrast, proper orientation and association with the ciliary vesicle was only observed in 20% of *talpid^2^* cells (Fig. 7A″-D″). Thus, we conclude that truncated C2CD3 in *talpid^2^* embryos impairs the ability of this protein to properly establish the transition fiber complex, which in turn affects intracellular ciliogenesis through failure of mother centriole/ciliary vesicle docking.

## DISCUSSION

The *talpid^2^* is an intriguing avian mutant that has been studied by the developmental community for many years (Abbott et al., 1959; Abbott, 1967; MacCabe and Abbott, 1974). Although disruptions in SHH signaling had been implicated in this mutant, there was a lack of understanding of the cellular and molecular mechanism for this phenotype. Furthermore, the genetic basis for this mutation was entirely unknown. We have previously suggested that defects in primary cilia exist within this mutant (Brugmann et al., 2010). Here, we confirm the aberrant structure and function of primary cilia in *talpid^2^* mutants and determine that SHH signaling is disrupted. Specifically, an increased amount of GLI3A in both the developing facial prominences and limb buds was observed in mutant embryos. We further identified C2CD3 as the presumptive locus containing the *talpid^2^* mutation via whole-genome sequencing and resequencing of multiple *talpid^2^* mutants. Resequencing identified a 19 bp deletion in *C2CD3* that causes a premature stop codon and produces a truncated protein in *talpid^2^* embryos. We hypothesize that this is the casual mutation for the *talpid^2^* phenotype. In addition to the 19 bp deletion, however, there is a missense mutation leading to an alanine-to-proline change at codon 782 consistently found among all *talpid^2^* alleles (supplementary material Table S5). Although this amino acid is not widely conserved in other vertebrate *C2CD3* genes, no other species contains proline at this codon. Therefore, we cannot eliminate the possibility that the Ala782Pro mutation, rather than the 19 bp deletion, leads to the mutant phenotype or, perhaps, a combination of both mutations. Reconciling the consequence(s) of this amino acid change is the focus of ongoing work.

### The SHH pathway is uncoupled in a prominence-specific manner in *talpid^2^* mutants

Our initial work identified increased expression of SHH pathway members in *talpid^2^* mutants (Brugmann et al., 2010). Herein, we performed more in-depth analyses using qRT-PCR, RNA-seq and *in situ* hybridization to individually analyze each facial prominence. Often, a direct relationship between ligand and pathway activity occurs; SHH ligand expression is coupled to an increase in pathway activity, as marked by an increase in *PTC* or *GLI1* transcription. Our analysis, however, suggests that this ‘coupling’ of the pathway is disturbed in *talpid^2^* mutants in a facial prominence-specific manner. For example, there is an increase of *SHH* ligand expression in the frontonasal prominence, but *PTC* expression is reduced. By contrast, the maxillary prominence has increased *SHH* ligand expression with no change in *PTC* expression (Fig. 3). From these data, we conclude that aberrant ciliogenesis uncouples the SHH pathway in *talpid^2^* mutants; however, the manner in which the pathway is perturbed is dependent upon the distinct molecular environment present in the individual facial prominences.

### Disruptions in GLI processing are conserved between ciliary mutants

Members of the GLI family of transcription factors have been well studied for their role in developmental processes (Hui and Angers, 2011). Defects in GLI activity have been associated with craniofacial anomalies in both humans (Greig cephalopolysyndactyly) (Vortkamp et al., 1991, 1992) and mice (Extra-toes) (Johnson, 1967). Our analysis of GLI processing during craniofacial development revealed a number of interesting findings. First, our data suggest that the *talpid^2^* mutation affects GLI2 differently from GLI3. GLI2 has a higher mobility in *talpid^2^* mutants, indicating a lack or reduction of post-translational modifications required for proper function of the protein (Fig. 4A; supplementary material Fig. S1A). GLI3, however, does not show any mobility shift or differential response to phosphatase treatment (Fig. 5A; supplementary material Fig. S1B). Thus, there could be a different cadre of proteins required for the processing of GLI2 than GLI3. Second, although examined in different tissues, the most-significant molecular defect in both *talpid^2^* and *talpid^3^* appears to be the increase in the levels of GLI3A (Fig. 5C) (Davey et al., 2006). An increase in GLI3A suggests a net-increase in pathway activity; however, data from studies of the *talpid^3^* suggest that there is a gain of SHH phenotype in the limb, yet a loss of SHH phenotype in the face (Buxton et al., 2004; Davey et al., 2006, 2007). By performing tissue isolations prior to western blotting, we confirmed that GLI processing was consistent among all facial prominences and the limb in *talpid^2^*. Thus, using GLI protein as an indicator of pathway activity, it can be concluded that there is a gain of SHH function in both the face and limb of *talpid^2^.* We hypothesize that this disruption is due to reduced ciliary function in *talpid^2^* embryos, rather than to a direct interaction with C2CD3 protein, as neither FLAG-*C2CD3^Ctrl^* nor FLAG-*C2CD3^ta2^* was able to pull-down GLI3 (supplementary material Fig. S4). It will be of great interest to determine whether *TALPID3* and *C2CD3* function in parallel, or as part of a greater complex necessary for GLI processing, centriolar docking or transition fiber formation.

### *talpid^2^* and *talpid^3^* are caused by distinct mutations within proteins that function in the same cellular compartment and signaling pathway

The talpid mutants have long been associated with one another based simply on presentation of some similar phenotypes. Limited distribution and resources have prevented a direct comparison of the two mutations. The *talpid^3^* mutant has been bred and maintained in the UK, whereas the *talpid^2^* mutant has remained in the USA. The *talpid^3^* mutation has been mapped to chromosome 5 and shown to be caused by a mutation in the *KIAA0568* gene, which encodes what is now called TALPID3 protein (Davey et al., 2006, 2007). Tickle and colleagues showed that the *KIAA0568* mutation responsible for the *talpid^3^* resulted in disorganized basal bodies that are unable to dock with the cell membrane and almost completely lack ciliary axonemes (Yin et al., 2009). Here, we show that the *talpid^2^* mutation is linked to a region on chromosome 1q within the avian ortholog of *C2CD3*. *C2CD3* was first identified via a forward genetic screen for recessive mutations affecting mouse embryonic development (Hoover et al., 2008). *hearty* and *talpid^2^* mutants both exhibit polydactyly, abnormal dorsal-ventral patterning and craniofacial anomalies, and both mutants have significantly increased levels of GLI3A (Abbott et al., 1959; Agarwala et al., 2005; Hoover et al., 2008). These phenotypic and molecular similarities strongly support our finding that a mutation in *C2CD3* is causal for the *talpid^2^* phenotype.

Although the exact function of C2CD3 is currently unknown, recent studies suggest C2CD3 is localized to the distal ends of both mother and daughter centrioles, and is required for the recruitment of centriolar distal appendage proteins (Jakobsen et al., 2011; Balestra et al., 2013; Ye et al., 2014). *In vitro* analysis further suggests that C2CD3 is required for recruiting the intraflagellar transport proteins IFT88 and IFT52 to the mother centriole, and is essential for ciliary vesicle docking to the mother centriole (Ye et al., 2014). Thus, our data, together with that of others, suggest that the C2CD3 protein regulates intracellular ciliogenesis by promoting the assembly of centriolar distal appendages that are crucial for docking ciliary vesicles and anchoring the centriolar complex to the cell membrane. This is an intriguing hypothesis, as the TALPID3 protein also reportedly functions to anchor the centriole to the membrane (Yin et al., 2009), perhaps via functioning within the transition fibers.

Future studies are needed to elucidate the exact role of C2CD3, primary cilia and GLI trafficking/processing in craniofacial development. The identification of avian *C2CD3* opens the possibility that *talpid^2^* may be a relevant model for human disease. Both the *talpid^2^* and *talpid^3^* mutations have provided new insights into craniofacial ciliopathies, which can be used to elucidate the pathways and regulatory mechanisms behind ciliopathic diseases. Interestingly, a number of human craniofacial ciliopathies are caused by mutations in proteins that localize to the distal centriole or proximal axoneme, including Meckel syndrome, Joubert syndrome, nephronophthisis and Senior-Loken syndrome (Reiter et al., 2012). The *C2CD3* ortholog in humans is in the HSA11q13.4 chromosomal region, in close proximity to the critical regions for other human ciliopathies, including Meckel-Gruber syndrome 2 (MKS; 603194), Joubert syndrome 2 (JBTS2; 608091) and Nephronophthisis 15 (NPHP15; 614845) (Roume et al., 1998; Hoover et al., 2008; Valente et al., 2010). Thus, the continued examination of the role of C2CD3 has direct relevance for the study of human diseases.

## MATERIALS AND METHODS

### Embryo preparation and measurement of facial prominences

*talpid^2^* heterozygous carriers were mated and eggs were collected. Embryos were incubated at 37°C for 2-7 days. Facial measurements were performed using Leica software. FNP length was defined as the superior aspect of the nasal pit to the distal tip of the FNP. FNP width was defined as the widest aspect of the distal FNP. Infranasal distance was the distance between the medial aspects of the nasal pits. Palatal patency was defined as the length of patency of the palatal shelves. *P*-values were determined using Student's two-tailed *t*-test with variance being unequal.

### Scanning and transmission electron microscopy

Facial and forebrain tissue were collected from control and *talpid^2^* HH20 embryos. Samples were fixed in 0.1 M sodium cacodylate (pH 7.4) containing 4% paraformaldehyde and 2% glutaraldehyde, re-fixed in 1% osmium tetroxide, dehydrated in ethanol, dried, mounted and sputter-coated with 20 nm of gold. Images were collected at 2.0 keV in a Zeiss Supra-35VP FEG.

For transmission electron microscopy, tissue was fixed with 3% glutaraldehyde, washed with sodium cacodylate buffer, post-fixed with 1% OsO_4_, processed through an ethanol gradient, washed in serial dilutions of plastic (Ladd Research, LX-112)/propylene oxide mixture and moved to 60°C for 3 days. The tissue was sectioned by Leica EM UC7 ultramicrotome at 100 nm and put onto 200 mesh copper grids.

### Chicken embryonic fibroblast isolation and transfection

Facial prominences and limbs were collected from HH25 control or mutant embryos, and were digested with 1 mg/ml collagenase at 37°C for 30 min. Cells were dissociated, washed in PBS and plated in DMEM with 10% fetal bovine serum and 50 U/ml penicillin-streptomycin. Chick embryonic fibroblasts (CEFs) were plated at 70% confluency and transfection was carried out using X-tremeGENE HP DNA transfection reagent (Roche) according to the manufacturer's protocol.

### Quantitative RT-PCR, RT-PCR and RNA-seq

Facial prominences were collected and pooled from ∼10 control or mutant embryos. RNA was extracted using TRIzol reagent (Invitrogen) and cDNA was made using SuperScript III (Invitrogen). SsoAdvanced SYBR Green Supermix (Bio-Rad) and a CFX96 Touch real-time PCR detection system (Bio-Rad) were used to perform quantitative real-time PCR. All the genes were normalized to GAPDH expression. Student's *t*-test was used for statistical analysis. *P*<0.05 was determined to be significant. Primers included: GAPDH F, CAACATCAAATGGGCAGATG; GAPDH R, AGCTGAGGGAGCT-GAGATGA; C2CD3 F, GAGGAAAGCAGTGAGGTTCTA AA; C2CD3 R, CTCCAGGATGGCAATTCG (amplicon size 782 bp).

For RNA-seq, individual facial prominences were harvested from control (*n*=8) and *talpid^2^* (*n*=8) embryos at HH25. RNAs were processed according to recommended procedures, using the Illumina TruSeq and Nugen Ovation RNA-Seq System V2 methods. Sequencing was carried out using the Illumina HiSeq 2000 system according to Illumina protocols. BAM files underwent whole-genome RNA-seq analysis using GeneSpring Version 12.5-GX-NGS. The reference chick genome was taken from Ensembl (4/9/2012). The Audic Claverie test (no correction) was used to identify differentially expressed genes between control and *talpid^2^* facial prominences with a *P*≤0.05. Heatmaps were generated by GeneSpring after performing the Pathway Analysis function. Raw data have been deposited in GEO under accession number GSE52757.

### *In situ* hybridization

*In situ* hybridization was performed using digoxigenin-labeled riboprobes, as described on the Gallus Expression in situ hybridization Analysis site (GEISHA) (Bell et al., 2004; Darnell et al., 2007).

### Immunohistochemistry

Immunostaining was performed according to standard protocols. Briefly, embryos were fixed in either 100% methanol or DENTS fixative. CEFs were fixed in 4% PFA. Samples were blocked with 10% serum (Millipore) for 30 min at room temperature and incubated with primary antibody at 4°C overnight and with Alexa Fluor 594Ms or 488Rb secondary antibodies (1:1000; A11020 or A11070; Invitrogen) for 1 h. Slides were stained with 4′,6-diamino-2-phenylindone (DAPI; 5 μg/ml; Invitrogen) and mounted (ProLong Gold, Invitrogen). Antibodies included rabbit anti-glutamylated-tubulin (1:500; AB3201, Millipore), mouse anti-γ-tubulin (1:1000; T6557, Sigma) and anti-FLAG (1:1000; F1804, Sigma). The percentage of primary cilium extension in CEFs was measured in at least 20 microscopic fields. The *P*-value was determined using Student's *t*-test.

### Western blot analysis

Facial prominences and limbs were dissected from HH25 control or *talpid^2^* embryos. Tissue was rinsed with ice-cold NaCl and sonicated in RIPA buffer containing protease and phosphatase inhibitors (Thermo Scientific, #87786). Protein concentration was measured by BCA protein assay (Thermo Scientific, #23227) and 30 μg protein was applied to a 6% SDS-PAGE gel. Antibodies used included anti-Gli2 antibody (1:500; R&D, AF3635), anti-Gli3 antibody (1:1000; R&D, AF3690) and anti-β-actin antibody (1:10,000; Abcam, ab8227).

### Nuclear fractionation

Facial prominences and developing limbs were dissected from HH 25 chicken embryos. Tissue was rinsed with PBS and digested with 2 mg/ml collagenase (Sigma, C2674). Nuclear fractionation was performed using NE-PER Nuclear and Cytoplasmic Extraction Reagents (Pierce, #78833).

### Phosphatase treatment

Total proteins (100 μg) from control facial prominences were collected and treated with phosphatase (New England Biolabs, P0753S) at 30°C for 1 h. Laemmli buffer was added and the sample was boiled at 95°C for 5 min before performing western blot analyses.

### Morpholino transfection

Control CEFs were grown to 80% confluency, trypsinized and resuspended in DMEM at a concentration of 1×10^7^/ml. Cells (3×10^6^ in 300 μl) were mixed with either 3 μl of 1 mM control-MO or with two separate C2CD3 translational MOs (C2CD3-MO1, 5′-CTGCATGGCTTCACCTCAAC-GCTT-3′; C2CD3-MO2, 5′-AACAGCCGCCCCGACGGGAGCATCT-3′) to a final concentration of 10 μM. Electroporation was performed using a geneZAPPER 450/2500 system at 950 μF and 200 V. After electroporation, 30 μl of the cells/well were plated on fibronectin-coated (5 μg/cm^2^) cover slips. Cells were serum starved for 48 h and immunostained. The percentage of ciliary extension in CEFs was measured in at least 20 microscopic fields. *P*-values were determined using Student's *t*-test.

### Genetic lines, sequencing and analysis

Embryonic samples used for this study were from four genetic lines: the developmental mutant-congenic inbred line talpid2.003 (also known as ta2.003) and its inbred (*F*>0.99) parent background line UCD003, as well as the *talpid^2^* mutation integrated onto a commercial white leghorn line (CWLL) and the outbred parental genetic background CWLL. The DNA from 10 known talpid2.003 mutants (−/−) and six inbred UCD 003 individuals were isolated and prepared for whole-genome sequencing. Samples were sequenced via NGS by DNA Landmarks (Saint-Jean-sur-Richelieu, Quebec, Canada) on the Illumina Hi-Seq platform. Reads were mapped to the reference chicken genome and polymorphisms (single nucleotide polymorphisms, SNPs, small insertion/deletions and structural rearrangements) were called using the CLC Genomics Workbench (CLC bio).

## Supplementary Material

Supplementary Material
