# Supplementary material for: The cellular and molecular etiology of the craniofacial defects in the avian ciliopathic mutant talpid2
Source: Development. 2014 Aug;141(15):3003–12. doi: 10.1242/dev.105924 (PMC4197679; doi:10.1242/dev.105924)
Supplement: Supplementary Material [file supp_141_15_3003__index.html]

The cellular and molecular etiology of the craniofacial defects in the avian ciliopathic mutant talpid2 — Supplementary Material 

# The cellular and molecular etiology of the craniofacial defects in the avian ciliopathic mutant *talpid2*

## DEV105924 Supplementary Material

**Files in this Data Supplement:**

- **Supplementary Material**
