# Supplementary material for: The cellular and molecular etiology of the craniofacial defects in the avian ciliopathic mutant talpid2
Source: Development. 2014 Aug;141(15):3003–12. doi: 10.1242/dev.105924 (PMC4197679; doi:10.1242/dev.105924)
Supplement: Supplementary Material [file supp_141.15.3003_DEV105924.pdf]

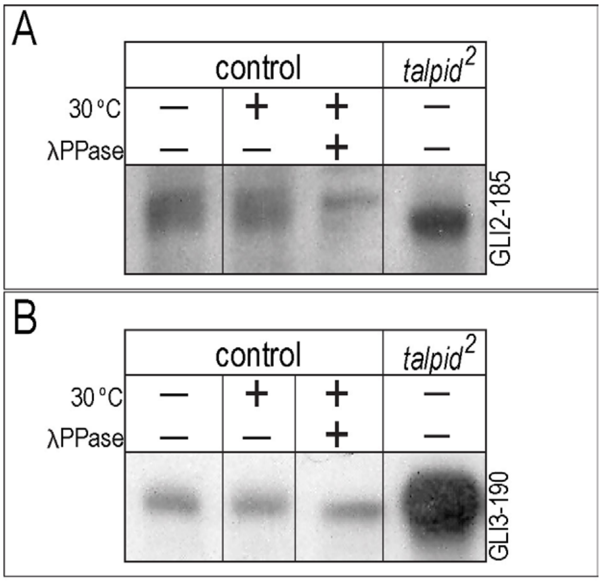

Figure S1. Phosphatase treatment of GLI2 and GLI3. (A) Phosphatase treatment of control full-length GLI2-185 did not shift mobility to that of the *talpid*^{2} GLI2-185. (B) Phosphatase treatment shifted GLI3-190 in both control and *talpid*^{2} embryos.

```
     GTGAAGGCGGGAGATGCTCCCGTCGGGGCGGCTGTTGCCGGGCAGCGGCGCGGCCTGAGG
     GAAGCGTTGAGGTGAAGCC
   1 ATGCAGCAGAGGAGGCCGCGGGCCTCAGCCCCAGCCCCGGGGGTCAGCCGGCGGCGTCGA 60
     M  Q  Q  R  R  P  R  A  S  A  P  A  P  G  V  S  R  R  R  R   20

      Exon2
  61 GCTCCCGGTGACATCCCGGCGTCCACCAGCCTGCCGCCGCTGGTGGAGGGCCCTCCGCGC 120
     A  P  G  D  I  P  A  S  T  S  L  P  P  L  V  E  G  P  P  R    40

 121 TGCGTCCTCTGCTGCACCGTGGGCAGAATCGTGTGGACGGCCGCCAAACCGCCCTCCGCC 180
     C  V  L  C  C  T  V  G  R  I  V  W  T  A  A  K  P  P  S  A    60

 181 GCCGTCCTCGTCCGCCTGAGGTGGTGGGGTGAGACCTCGGACGGTACCGTGTTCCACCCC 240
     A  V  L  V  R  L  R  W  W  G  E  T  S  D  G  T  V  F  H  P    80

 241 TCCTCAAAGCCGGGGCAGGCGGCGGGCAGCAGCCGGTACACCGTGCGGTGCGGGCCTCGG 300
     S  S  K  P  G  Q  A  A  G  S  S  R  Y  T  V  R  C  G  P  R   100

                              Exon3
 301 CAGCTCACGGCCTACCTCACAGATATGGGCATGCTTGTCCTGGAGGTCCTGACCAAACCT 360
     Q  L  T  A  Y  L  T  D  M  G  M  L  V  L  E  V  L  T  K  P   120

 361 GACCGTCTTCCAGTTGGCAGAGTGCAGATCACCGAATTGTCCCAGCTCTCTCCCAGCCAT 420
     D  R  L  P  V  G  R  V  Q  I  T  E  L  S  Q  L  S  P  S  H   140

                Exon4
 421 CCCATCAGTGGGTTTTTCACCATAGTTTCACCAACAGCTGATAAACTGGGAGAGCTACAG 480
     P  I  S  G  F  F  T  I  V  S  P  T  A  D  K  L  G  E  L  Q   160

 481 GTTTCACTTGTTCTCGAACCTCTATCAGACACATACAAAACCAGCAGCTCTGTCCCAACC 540
     V  S  L  V  L  E  P  L  S  D  T  Y  K  T  S  S  S  V  P  T   180

 541 ACAGCTACAAGTTCAGATATGACTCCTGCTCCATCGCAGCAAATGCAGACCACTCCTCCT 600
     T  A  T  S  S  D  M  T  P  A  P  S  Q  Q  M  Q  T  T  P  P   200

                                             Exon5
 601 GGCCAAGAGCCTGTTAACAAAAGCAGGGTCACGACTCCCAGTTGGGGAAAGGACCATTCA 660
     G  Q  E  P  V  N  K  S  R  V  T  T  P  S  W  G  K  D  H  S   220

 661 TTCTTTCAAGAGAACTCTGAAAACATAAAGGATGTTTCCTCTGCTTCCCACCACCGCCTC 720
     F  F  Q  E  N  S  E  N  I  K  D  V  S  S  A  S  H  H  R  L   240

 721 ATTTTACCAGATGAACATTTGAAAGTGAATCCTTTGAGTCAGCAGTCAGTATCATTCCCC 780
     I  L  P  D  E  H  L  K  V  N  P  L  S  Q  Q  S  V  S  F  P   260

 781 AGCAGCACTGATCCTGAAGCACCCCTCAGTACAAACACACACGTGTTCTCCTTGCACAAC 840
     S  S  T  D  P  E  A  P  L  S  T  N  T  H  V  F  S  L  H  N   280

                                 Exon6
 841 CCAACTACAAAAGACCTGCTTTCAGCTCTTTTGGATCAAGGCAATAAACTCCGTGATGCC 900
     P  T  T  K  D  L  L  S  A  L  L  D  Q  G  N  K  L  R  D  A   300

 901 ATGGTTGCTTCTGCAATGAAGTCCAGCCCAGACATGGAGATTGAATTGAATGAAGTGCCT 960
     M  V  A  S  A  M  K  S  S  P  D  M  E  I  E  L  N  E  V  P   320

                                          Exon7
 961 CCTTTTGTAAAGCCAGATGGTTTCAGAGCAGCAGCAAAGAGCCCAAAAGGCTTGAACTCA 1020
     P  F  V  K  P  D  G  F  R  A  A  A  K  S  P  K  G  L  N  S    340

1021 ACTTTTATGCCTCCTCCTGAGGATACCCATCTCTTCACATCTGAAGTCACAGGTCAGCAA 1080
     T  F  M  P  P  P  E  D  T  H  L  F  T  S  E  V  T  G  Q  Q    360

                                                    Exon8
1081 TTAGACCTCAACTCTGAGGAGAGAGCAATACAGTTACTGTTGGGCAGTGCTGATTTATCT 1140
     L  D  L  N  S  E  E  R  A  I  Q  L  L  L  G  S  A  D  L  S    380

1141 CCTGTGCATTTCTGGGATCAGCCAGGTTCCCTTCTGGATTCTCTCTCTGTTGGAAGTGAG 1200
     P  V  H  F  W  D  Q  P  G  S  L  L  D  S  L  S  V  G  S  E    400

1201 GTGTATGACAGTGAACTCAATGATCCCCACTATGACCAGAGTCTGCTAGAAAGTCTTTTT 1260
     V  Y  D  S  E  L  N  D  P  H  Y  D  Q  S  L  L  E  S  L  F    420

                 Exon9
1261 TACACGACACCTAAATCAGATTCCAGTACAAGTGATTTCCTCAGTGATGAAGATGTTAAC 1320
     Y  T  T  P  K  S  D  S  S  T  S  D  F  L  S  D  E  D  V  N    440

1321 CCTTCCAAAAAAGTAAGCAAAACAAAAACTGTGAAGAAAGCTGATTCTGTGAATGCACAG 1380
     P  S  K  K  V  S  K  T  K  T  V  K  K  A  D  S  V  N  A  Q    460

                                                          Exon10
1381 AAGGATTCCACTGCCTTAGATCAGGATCAGAAGGTGACAGAAATTAAACCCAGCTGCTCT 1440
```

Figure S2. cDNA and amino acid sequence of chicken C2CD3.

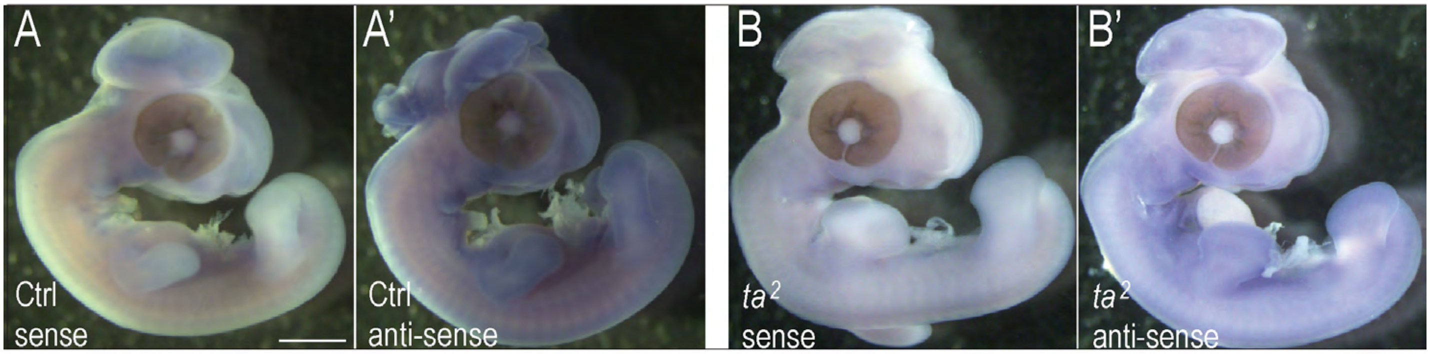

Figure S3. *C2CD3* is expressed in whole chicken embryos. (A-B') *In situ* hybridization for *C2CD3* in day 4 whole embryos. Scale bar represents 500 µm.

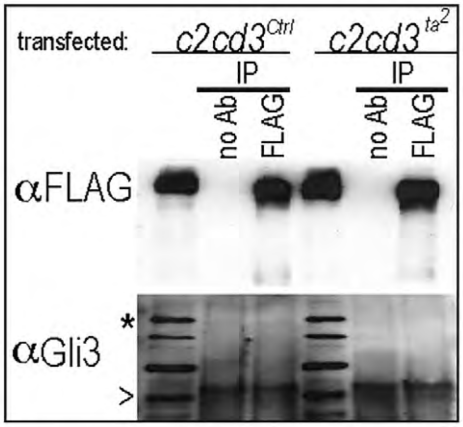

Figure S4. C2CD3 does not interact with GLI3. CEFs were transfected with either p3XFLAG-c2cd3^{Ctrl} or p3XFLAG-c2cd3^{ta2} and immunoprecipitation was performed with anti-GLI3. * indicates GLI3-190, > indicates cleaved GLI3-83.

Table S1. Densitometry readings of GLI2 and GLI3 nuclear fraction

		GLI2			GLI3			LAMIN	
		raw #	normalized		raw#	normalized		raw #	normalized
FNP	ctrl	1926.882	1		3982.368	1		2948.882	1
	TA2	2688.489	1.395253575		16254.154	4.081529884		4076.276	1.382312348
MXP	ctrl	4366.518	1		6927.711	1		3294.225	1
	TA2	4703.43	1.077158047		19972.267	2.882953258		3812.296	1.157266428
MNP	ctrl	2385.305	1		3486.794	1		4547.205	1
	TA2	4756.903	1.994253565		12053.296	3.456842016		9019.539	1.983534721

Table S2.

Download Table S2

Table S3.

Download Table S3

Table S4. Variant frequency in ta2.003 candidate intervals

candidate ta-2 region Galgal4 coordinates	size Mb	UCD003 SNP	UCD003 DIP	tal2-specific SNP	tal2-specific DIP	UCD003 SNP/Mb	tal2-specific SNP/Mb	fixed tal2-specific SNP	fixed tal2-specific SNP/Mb	fixed ta-2/UCD003 SNP ratio	tal2/UCD003 DIP ratio
chr1:115764249-118327843	2.56	25484	1407	2575	335	9955	1006	752	294	0.030	0.24
chr1:193748573-195187547	1.44	15300	658	2819	238	10625	1958	1550	1076	0.101	0.36
chr10:8513002-10170824	1.66	14620	815	1134	151	8807	683	314	189	0.021	0.19
chr10:11552743-12453463	0.90	11022	627	730	95	12247	811	89	99	0.008	0.15
chr15:10301074-11866661	1.57	17508	857	125	40	11152	80	59	38	0.003	0.05

Variant frequencies are shown (total and per Mb) for UCD003 and ta2.003-specific. Fixed refers to those variants found in all ta2.003 reads that likely include fewer false positives. DIP, deletion/insertion polymorphism.

Table S5. Coding variants in C2CD3

mRNA coord rel to ATG 89	Coordinate (Galgal4) 194883819	Reference C	ta2.003 G	GGA1 Reference Amino Acid Change Ser30Thr	variant in ta2.003
167	194883741	T	C	Lys56Arg	ta2.003
957	194879146	C	T	synonymous	ta2.003
2178	194867809	G	A	synonymous	ta2.003
2325	194867662	G	A	synonymous	ta2.003
2344	194867643	C	G	Ala782Pro	ta2.003
2346	194867641	A	G	synonymous	ta2.003
4049	194858002	C	T	Gly1350Asp	ta2.003
4595	194856869	G	A	Pro1532Leu	ta2.003
4598	194856866	G	A	Thr1533Ile	ta2.003
4674	194856790	C	A	Gln1558His	ta2.003
5870	194844961	C	T	Gly1957Asp	ta2.003
6570	194844261	T	C	synonymous	ta2.003
6626-6644	194844184-194844202		19 bp deletion	deletes a.a. from 2210-2330	ta2.003
6648	194844180	A	G	synonymous after frameshift/stop	ta2.003
6767	194842820	A	G	Val2256Ala after frameshift/stop ta2.003	
1022	194877027	G	T	Thr341Asn	ta2.003 and UCD003
1194	194875767	T	C	synonymous	ta2.003 and UCD003

Exonic variants confirmed by whole genome sequencing and resequencing in C2CD3.
